# Study on the network of postoperative symptoms and its influencing factors in esophageal cancer patients

**DOI:** 10.3389/fonc.2026.1702233

**Published:** 2026-02-27

**Authors:** Jing Zhao, Yina Liu, Bingbing Xiao, Tingting Tan, Yiting Yin, Yang Hu, Li Li

**Affiliations:** College of Nursing, North Sichuan Medical College, Sichuan, China

**Keywords:** chokingsensation, core symptoms, esophageal cancer, fatigue, influencing factors, network analysis

## Abstract

Esophageal cancer(EC) is a common and highly invasive malignant tumor of the digestive tract, for which surgery remains a key treatment modality. Postoperative patients frequently experience multiple coexisting physical and psychological symptoms. These symptoms are interrelated, forming a complex network that can substantially affect rehabilitation and health-related quality of life. Current symptom management often relies on traditional classification methods, which may be insufficient to elucidate the intrinsic relationships and core mechanisms among symptoms. Network analysis offers a new approach for identifying core symptoms, bridging symptoms, and key influencing factors. This study employs such methods to explore the relationships among postoperative symptoms in esophageal cancer, with the aim of providing evidence to support precise interventions and to enhance the effectiveness of symptom management.

**Objective:**

To construct a symptom network for post-operative esophageal cancer patients, identify core and bridging symptoms, and explore influencing factors, thereby providing references for precise and efficient symptom management.

**Methods:**

A convenience sampling method was used to select 263 patients with esophageal cancer undergoing surgery for investigation, and general information questionnaires and esophageal cancer perioperative symptom assessment scales were used. Univariate analysis and multivariate linear regression analysis were used to identify the influencing factors: bridge symptoms between core symptoms and symptom clusters were identified based on network analysis.

**Results:**

After surgery, esophageal cancer patients exhibited three symptom clusters: eating-related symptoms, pain and fatigue-related symptoms, and somatic-psychological symptoms, with a cumulative variance contribution rate of 67.841%. In the symptom network, fatigue (strength centrality,*rs* = 2.10) was the strongest core symptom, while a choking sensation (*rs* = 0.10) had the highest bridge strength among the bridge symptoms. The univariate analysis of results showed that gender, educational level, marital status, employment status, medical payment method, per capita monthly household income, pathological type, tumor location, disease duration, and whether postoperative or pre-discharge gastrointestinal nutrition tubes were used were not statistically significant (P>0.05). However, age, presence or absence of other chronic diseases, surgical method, treatment method, postoperative hospital stay, and tumor stage were statistically significant (P<0.05).

**Conclusion:**

Fatigue is the primary symptom for esophageal cancer patients post-surgery; Healthcare providers can use symptom network analysis to promptly identify core symptoms, bridge symptoms, and influencing factors, thereby formulating precise and effective intervention measures to improve symptom management efficiency and reduce the postoperative symptom burden on esophageal cancer patients.

Esophageal cancer (EC) is a common type of malignant tumor in the digestive tract. Data indicates that approximately 367,700 new cases of esophageal cancer are diagnosed annually in China, with 187,500 deaths, accounting for 6.9% of all cancer-related deaths (1). Currently, surgery is the primary treatment for esophageal cancer, supplemented by chemotherapy or immunotherapy. Postoperative care has become a crucial part of the treatment for resectable esophageal cancer, significantly improving surgical resection rates and patient outcomes (2).However, patients often experience a range of physical and psychological discomforts. Before surgery, the tumor can compress the esophagus, causing difficulty in swallowing and eating. This may negatively impact wound healing and postoperative recovery. An important aspect of preoperative rehabilitation is the optimization of the nutritional status of these patients. After surgery, changes in the esophageal anatomy can lead to symptoms such as reflux and diarrhea (3). These discomforts persist post-surgery, leading to anxiety, depression, and other negative emotions (4), which can significantly impact the patient’s quality of life. These symptoms typically do not appear in isolation but occur simultaneously and are closely linked, forming a symptom cluster. Research reports indicate that (5, 6), the symptom cluster can consist of two or more interconnected symptoms, and there is a synergistic effect among the symptoms within the same group, which adversely affects the patient’s physiological function, quality of life, and prognosis. The study mainly focused on the clinical characteristics and interventions of esophageal cancer (7, 8), while relatively few studies were conducted on the internal relationship between symptoms. The symptom network can further identify core symptoms (9) by quantitatively analyzing the complex relationship between symptoms and visualizing symptoms. According to the symptom network theory, core symptoms play a key role in the synergistic effect of symptoms, and effectively solving the problem of core symptoms can better reduce the overall symptom burden of patients (10).Studies have shown that core symptoms persist in the symptom cluster, and these core symptoms can be alleviated through high-intensity intervention, while helping to reduce or eliminate other related symptoms, thus improving the management efficiency of the symptom cluster (11). In recent years, it has been gradually applied to the symptom management of cancer patients (12, 13).By identifying core symptoms and bridge symptoms, this study explored the influencing factors of core symptoms in esophageal cancer patients after surgery, explored the role target of precision intervention, and provided a reference for clinical medical staff to develop more accurate and efficient symptom management programs.

## 1 Object and methods

### 1.1 Selection of research subjects

Patients who underwent radical esophageal cancer surgery for the first time in thoracic surgery departments of two grade A tertiary hospitals in Nanchong City, Sichuan Province from February 2025 to May 2025 were selected as research subjects. Inclusion criteria: patients diagnosed with esophageal cancer by fiberoptic esophagogastric endoscopy; patients who underwent radical surgery for esophageal cancer for the first time; patients aged 18 or older; patients who could complete the questionnaire independently; and patients who were informed of the diagnosis and agreed to participate in the survey. Exclusion criteria: severe cognitive impairment and language expression defects; patients who have participated in other clinical studies; patients with severe heart, lung, kidney and other important organ diseases and other malignant tumors. Exclusion criteria: Participants who withdraw for various reasons during the study; patient deaths; and participants who need to be terminated due to changes in disease treatment or condition. According to Epskamp and Fried’s network analysis tutorial (14, 15), for a partially correlated network with P nodes, the sample size should be at least P (P-1)/2 + P to achieve sufficient statistical power. This method of sample size calculation has been applied in numerous domestic symptom network studies (16). In this study, P represents the 19 symptoms (nodes). Considering a 10% dropout rate, the final sample size was determined to be at least 212 cases. The study has been approved by the ethics committee (approval number: 2025ER22-1).

### 1.2 Research tools

#### 1.2.1 General information survey form

The survey includes demographic data of esophageal cancer patients, such as age, gender, education level, marital status, employment status, payment methods for medical expenses, and average monthly household income, as well as disease-related information, including the pathological type, tumor location, tumor stage, disease duration, presence or absence of other chronic diseases, treatment method, surgical approach, postoperative use of gastrointestinal feeding tubes, pre-discharge use of gastrointestinal feeding tubes, and length of hospital stay.

#### 1.2.2 Esophageal cancer perioperative symptom assessment scale

The scale was developed by Yang Meng et al (17) based on the M.D. Anderson Symptom Inventory (MDASI) and added an esophageal cancer module with Chinese characteristics. The scale consists of two parts. Part 1 consists of 19 common symptom items, including 13 core symptoms and 6 esophageal cancer specific symptoms (dysphagia, weight loss, choking, foreign body sensation, substernal discomfort, reflux), to assess the severity of symptoms in the past 24 hours; The second part evaluates the impact of the 19 symptoms on six aspects of daily life: general activities, work, emotions, walking, interpersonal relationships, and enjoyment of life. Each symptom is rated on a scale from 0 to 10, with 0 indicating ‘no symptoms’ and 10 indicating ‘the most severe symptoms.’ The scale has good reliability and validity, making it suitable for assessing the perioperative symptoms of esophageal cancer patients in China. The Cronbach’s α coefficient for the first part of the scale is 0.883.

### 1.3 Data collection method

The research team, with the department’s consent, distributed questionnaires post-surgery. The researchers conducted one-on-one interviews with eligible patients, using standardized instructions to explain the study purpose and how to complete the questionnaire. Patients completed the questionnaire independently. The completed questionnaires were collected and verified on-site to prevent errors or omissions, ensuring the data’s completeness and accuracy. A total of 270 questionnaires were distributed, but 7 patients were excluded due to severe chronic diseases that prevented surgery. Ultimately, 263 valid questionnaires were recovered.

### 1.4 Statistical methods

In this study, SPSS25.0 software was used to analyze the incidence, severity, and factors influencing core symptoms of esophageal cancer patients. For quantitative data with a normal distribution, the mean ± standard deviation (x ± s) was used to represent the data. Inter-group comparisons were conducted using two independent samples t-tests and Anova, Quantitative data that did not conform to the normal distribution were expressed by median and quartiles M (P25, P75). Mann-Whitney U test was used for inter-group comparison. Multivariate linear regression analysis was used to explore the influencing factors of core symptoms. P <0.05 was considered statistically significant. Select symptoms with an occurrence rate greater than 20% for symptom cluster extraction and network analysis (18). Use principal component analysis combined with maximum variance orthogonal rotation to extract symptom clusters, ensuring that each factor includes at least two symptoms with a factor loading greater than 0.4 (19). The symptom network is constructed using the qgraph package in R software. Symptoms are represented as nodes, and the lines connecting them represent the edges of the network, where thicker and darker lines indicate stronger correlations between symptoms. The centrality indicators in the symptom network, also known as core symptoms, reflect the number, intensity, and closeness of connections between a specific symptom and other symptoms. Altering a symptom with high centrality can significantly impact many other symptoms. The centrality indicators in the network model include Strength, Closeness Centrality, Betweenness Centrality, and Expected influence. The strength is the sum of the absolute values of the correlation coefficients for each edge. The higher the strength, the more strongly the symptom influences other symptoms; the closeness refers to the average shortest path length from one symptom to all others; the mediation refers to the frequency of a symptom appearing on the shortest paths to all other symptoms. A higher value of this indicator suggests that the symptom is more likely to act as a bridge symptom; The expected impact is a measure of the total effect of one symptom on other symptoms through its direct connection. It is a key indicator in the symptom network, similar to intensity, and measures the direct and indirect effects of one symptom on other symptoms. Research indicates that the intensity indicator is more stable compared to other indicators (20). Therefore, this study uses the intensity indicator to identify core symptoms. The bootnet package was used to detect the statistical correlation between the centrality indicators of the new dataset and the original dataset after reducing the sample size in the network. The stability of the centrality indicators was assessed by calculating the Correlation Stability coefficient, which should be at least 0.25. The 95% confidence interval for the edge weights was calculated using the non-parametric bootstrap method, with a narrower confidence interval indicating a more precise estimation of the edge weights (21).

## 2 Results

2.1

In this study, the age of 263 patients with esophageal cancer ranged from 51 to 90 (68.47 ± 6.57). Other data are shown in **Table 1**.

**Table 1:** General information of esophageal cancer patients (n=263).

Item	Classify	Examples	Percentage%
gender	Man	216	82.13
	Woman	47	17.87
age	<60	48	18.25
	60~80	199	75.67
	>80	16	6.08
educational background	Primary school or below	216	82.13
	Junior middle school	36	13.69
	High school/vocational high school	9	3.42
	College degree or above	2	0.76
marital status	Married	242	92.02
	Single	1	0.38
	Divorced	2	0.76
	Widowed	18	6.84
Employment status	Unemployed	243	92.4
	Retired	20	7.60
Medical payment methods	Urban Employee Basic Medical Insurance	17	6.46
	Urban and Rural Resident Basic Medical Insurance	246	93.53
Per capita monthly household income	<1000	190	72.24
	1000~3000	39	14.83
	3000~5000	28	10.65
	>5000	6	2.28
Pathological type	Squamous cell carcinoma	244	92.78
	Adenocarcinoma	13	4.94
	Other	6	2.28
Site of tumor	Upper segment of esophagus	41	15.59
	Mid-esophagus	137	52.09
	Lower esophagus	75	28.52
	Upper middle section	4	1.52
	Lower middle section	6	2.28
course of disease	≤6 months	235	89.35
	6~12 months	13	4.94
	≥12 months	15	5.7
Whether there are other chronic diseases	Yes	179	68.06
	No	85	32.32
Surgical method	Thoracoscopic laparoscopy Open-heart surgery	246 17	93.53 6.46
Treatment method	Neoadjuvant therapy followed by surgery	160	60.84
	Surgical only	103	39.16
Postoperative gastrointestinal feeding tube (Yes/No)	Yes	259	98.48
	No	4	1.52
Gastrointestinal feeding tube before discharge (Yes/No)	Yes	12	4.56
	No	251	95.44
Postoperative hospital stay	10~20days	247	93.92
	>20days	16	6.08
Tumor staging	1	63	23.95
	2	92	34.98
	3	100	38.02
	4	8	3.04

Due to the advanced mean age of the study participants (68.47 ± 6.57 years) and the fact that 92.4% were retired, the category of ‘employed’ was not included in the employment status classification. The term ‘unemployed’ in this context refers to patients below retirement age who were not working due to health reasons or other circumstances.

### 2.2 Postoperative symptom incidence and symptom cluster extraction in esophageal cancer patients

The top 5 symptoms experienced by esophageal cancer patients upon discharge were weight loss (92.39%), restless sleep (84.41%), shortness of breath (84.41%), fatigue (83.79%), and pain (81.75%). The top 5 symptoms in terms of severity were restless sleep (5 (4,5) points), fatigue (5 (3,5) points), pain (5 (3,5) points), weight loss (4 (3,4) points), and difficulty swallowing (3 (3,4) points).An exploratory factor analysis was conducted on symptoms with an incidence rate greater than 20%. The suitability test for factor analysis showed a KMO value of 0.667 and a Bartlett’s test P-value of <0.001, indicating that the data were suitable for factor analysis. The results indicated that the number of symptom clusters among esophageal cancer patients at discharge was three, with a cumulative variance contribution rate of 67.841%. Postoperative symptom clusters were named the eating-related symptom cluster, the pain-fatigue-related symptom cluster, and the somatic-psychological symptom cluster. See **Table 2** for details.

**Table 2:** Postoperative symptom incidence, severity and symptom group extraction results of esophageal cancer patients (n=263).

Symptom items				Factor loading		
symptom	Number of cases (percentage%)	Severity [points, m (p25, p75)]	Mean	Eat related symptom cluster	Pain-fatigue related symptom cluster	Somatic-psychological symptoms
pain	215(81.75)	5(3, 5)	3.95		0.847	
weary	223(84.79)	5(3, 5)	3.86		0.851	
nausea						
Insomnia	222(84.41)	5(4, 5)	3.95		0.916	
vexed	200(76.04)	3(2, 4)	2.56			0.877
Dyspnea	222(84.41)	3(2, 3)	2.57			0.583
forgetful						
anorexia						
sleepy	24(9.13)	0(0, 0)	0.19			
dry						
sad	192(73.00)	3(0, 4)	2.43			0.892
vomit						
numb	16(6.08)	0(0, 0)	0.13			
dysphagia	211(80.23)	3(3, 4)	2.83	0.862		
Body weight loss	243(92.39)	4(3, 4)	3.49			
Achoking sensation	158(60.08)	3(0, 4)	2.25	0.717		
foreign body sensation	214(81.37)	3(3, 4)	2.85	0.890		
Discomfort behind the sternum	20(7.60)	0(0, 0)	0.16			
palirrhea	22(8.37)	0(0, 0)	0.17			

### 2.3 Network analysis of postoperative symptoms in esophageal cancer patients

**Figure 1:**
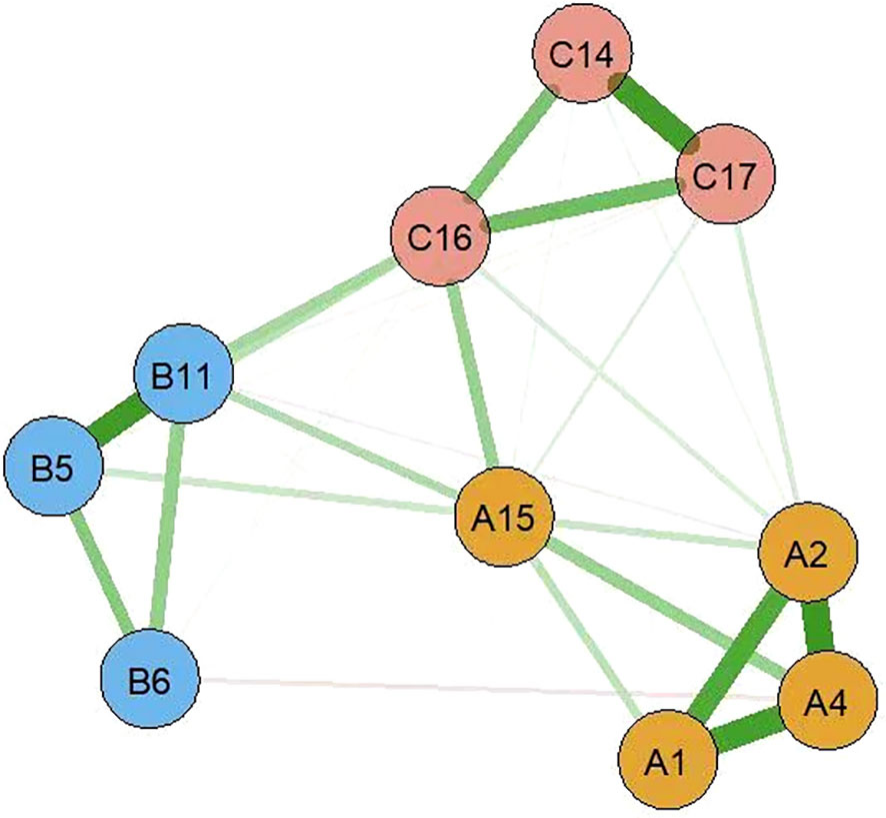
Symptom network diagram.

**Figure 2:**
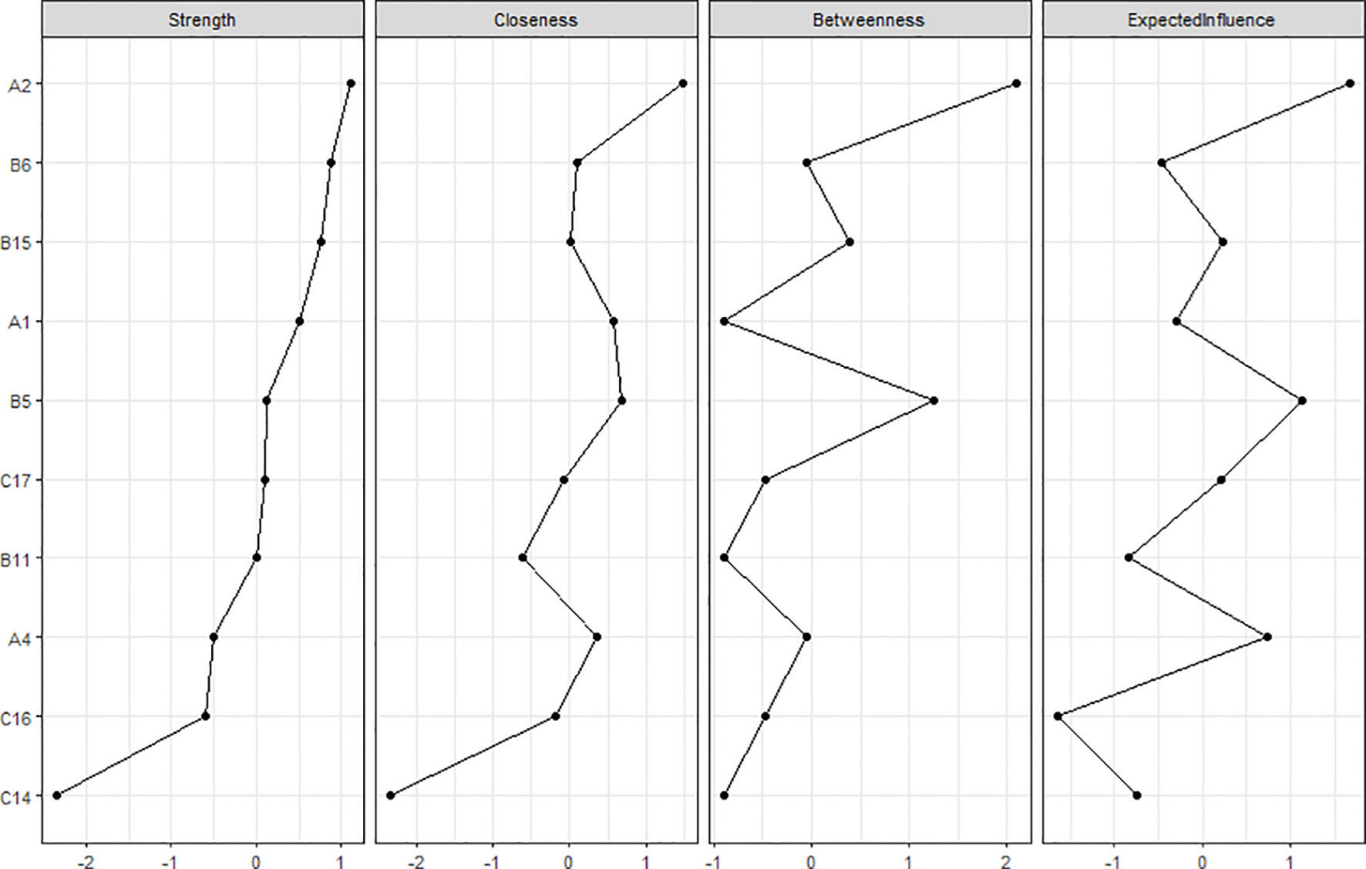
Centrality index of network nodes.

**Figure 3:**
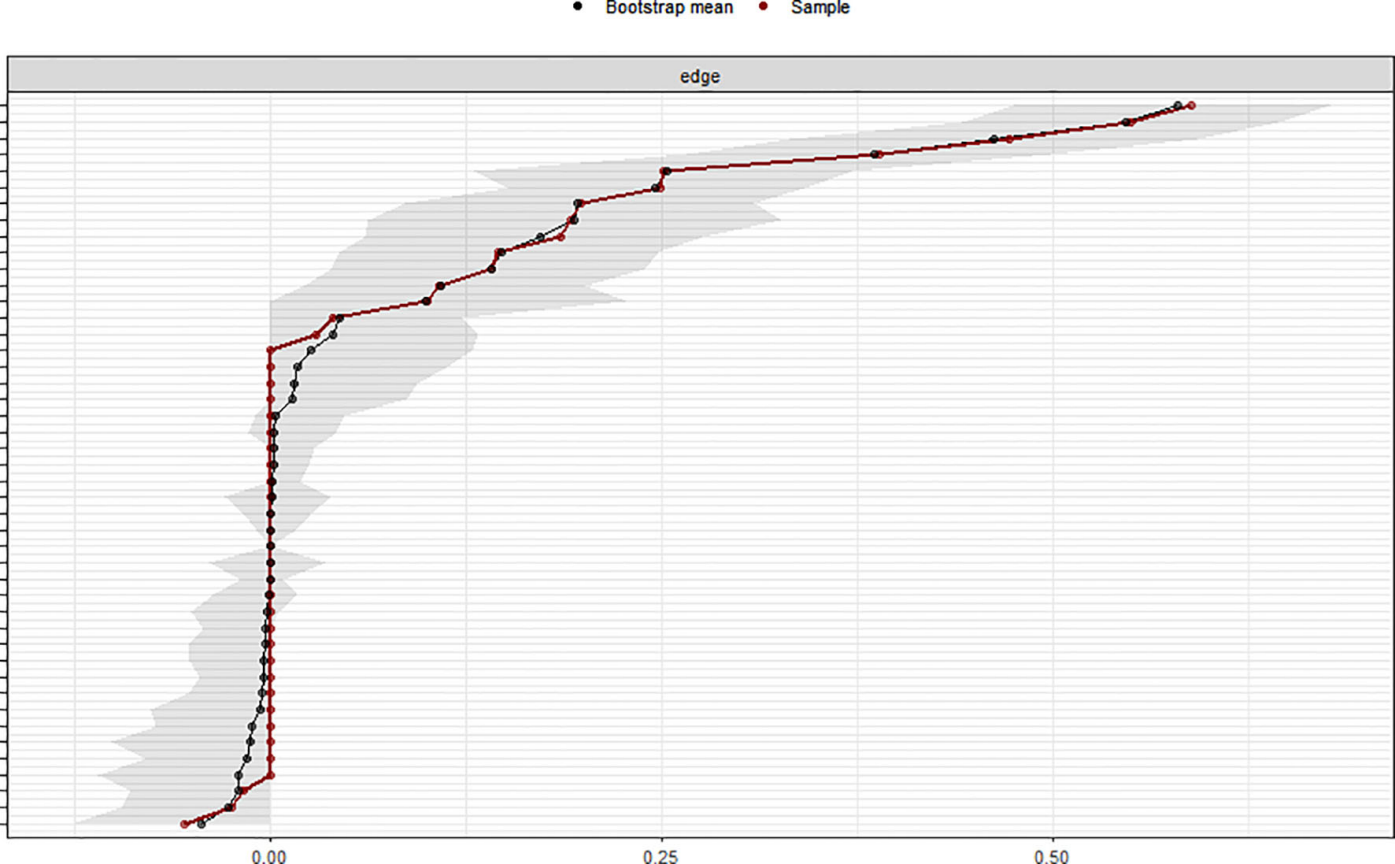
Stability test of symptom network.

**Figure 4:**
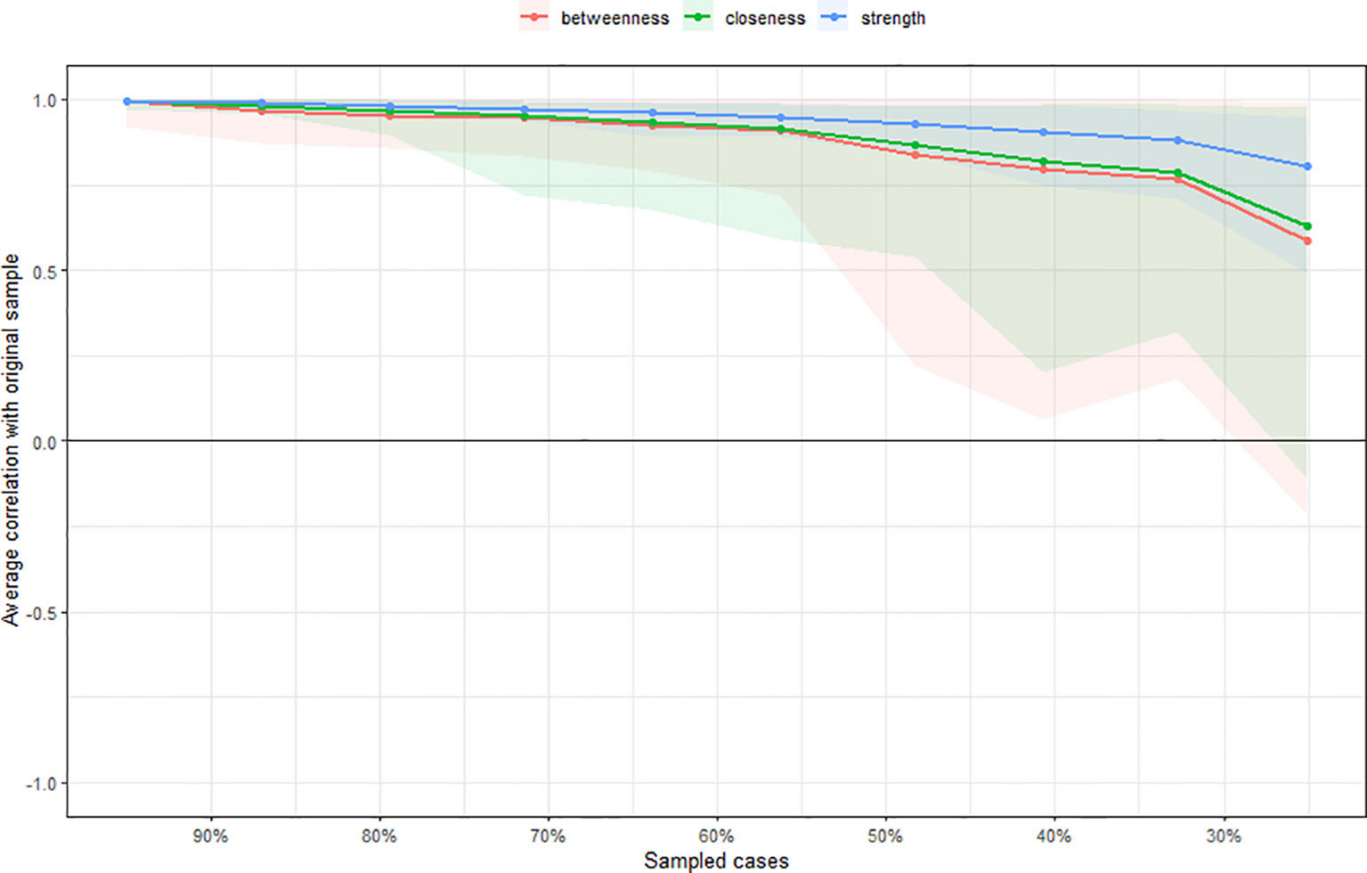
Edge weight accuracy test of symptom network.

**Figure 5:**
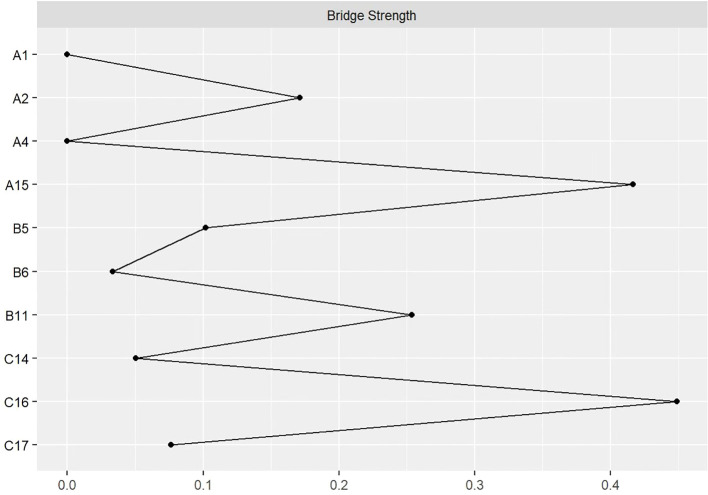
Bridge strength of the symptom network.

In the symptom network, thicker and darker edges indicate stronger correlations between symptoms, as shown in **Figure 1**. The symptom with the highest intensity centrality postoperatively is fatigue (*rs* = 2.10), followed by significant centrality in weight loss (Closeness Centrality, *rc* = 0.025), as illustrated in **Figure 2**.The 95% confidence interval of the edge weight (gray area) is relatively small, indicating good network accuracy. See **Figure 3**. The stability coefficients for strength correlation are 0.673, closeness centrality correlation is 0.437, and intermediary centrality correlation is 0.361. In this study, the stability coefficients for the strength of the symptom network are all above 0.25, as shown in **Figure 4**. The bridge strength of choking sensation (*rs* = 0.10) is the highest among bridge symptoms, as illustrated in **Figure 5**.

## 3 The results of the univariate analysis of factors affecting postoperative fatigue in esophageal cancer patients

Gender, educational level, marital status, employment status, medical payment method, average monthly household income, pathological type, tumor location, disease duration, and the presence or absence of a gastrointestinal feeding tube before and after discharge were not statistically significant (P>0.05). However, age, presence or absence of other chronic diseases, surgical method, treatment method, postoperative hospital stay, and tumor stage were statistically significant (P<0.05). See **Table 3**.

**Table 3:** Univariate analysis of postoperative fatigue in esophageal cancer patients.

Variable	Examples	Fatigue rating	t(F)value	P value
sex			0.163	0.687
man	216	3.88 ± 1.85		
woman	47	3.76 ± 2.04		
age			3.555	0.008
<60	48	4.18 ± 1.61		
60∼80	199	3.87 ± 1.91		
>80	16	2.75 ± 2.04		
degree of education			0.652	0.582
Primary school and below	216	3.89 ± 1.91		
Primary school and below	36	3.88 ± 1.80		
High school/vocational high school	9	3.00 ± 1.80		
College degree or above	2	4.00 ± 0.70		
marital status			0.316	0.814
married	242	3.88 ± 1.88		
single	1	3.00		
divorced	2	4.50 ± 0.70		
Widowed	18	3.55 ± 1.89		
Active status			2.438	0.120
unemployed	243	3.17 ± 1.77		
retire	20	3.91 ± 1.89		
Medical payment methods			0.578	0.448
Medical insurance for urban workers	17	3.53 ± 1.87		
Medical insurance for urban and rural residents	246	3.89 ± 1.89		
Per capita monthly household income			0.833	0.477
<1000	190	3.95 ± 1.86		
1000~3000	39	3.58 ± 2.12		
3000~5000	28	3.53 ± 1.89		
>5000	6	4.33 ± 0.81		
Pathological type			1.692	0.186
Squamous cell carcinoma	244	3.76 ± 1.90		
Adenocarcinoma	13	4.22 ± 1.80		
other	6	4.42 ± 1.80		
Site of tumor			0.298	0.879
Upper segment of esophagus	41	4.00 ± 1.85		
Mid-esophagus	137	3.84 ± 1.94		
Lower esophagus	75	3.86 ± 1.82		
Upper middle section	4	4.25 ± 0.95		
Lower middle section	6	3.16 ± 2.48		
course of disease			0.681	0.507
≤6months	235	3.44 ± 1.79		
6~12months	13	3.72 ± 2.02		
≥12months	15	3.46 ± 2.44		
Whether there are other chronic diseases			3.942	0.048
yes	179	3.70 ± 1.92		
no	85	4.20 ± 1.77		
modus operandi			4.415	0.037
Thoracoscopic laparoscopy	246	3.93 ± 1.86		
Open-heart surgery	17	2.94 ± 2.04		
Therapymethod			4.241	0.040
Preoperative neoadjuvant therapy + surgery	161	4.16 ± 1.67		
Surgery alone	103	3.67 ± 1.99		
Whether there is a gastrointestinal feeding tube after surgery			1.295	0.256
yes	259	3.80 ± 1.95		
no	4	4.13 ± 1.56		
Whether there is a gastrointestinal feeding tube before discharge			0.412	0.522
yes	12	3.53 ± 1.76		
no	251	3.88 ± 1.89		
Number of days in hospital after surgery			5.389	0.021
10~20days	247	3.93 ± 1.87		
>20days	16	2.81 ± 1.86		
Tumor staging			2.878	0.037
1	63	3.44 ± 1.78		
2	92	3.95 ± 2.01		
3	100	4.15 ± 1.75		
4	8	2.75 ± 2.31		

## 4 Multivariate linear regression analysis of postoperative fatigue in esophageal cancer patients

The variables with statistically significant differences identified in the univariate analysis were used as independent variables, and the postoperative fatigue score was used as the dependent variable for the multivariate linear regression analysis. The results showed that age and treatment method significantly influenced the severity of postoperative fatigue in esophageal cancer patients (P<0.05). See **Table 4**.

**Table 4:** Multivariate linear regression analysis of postoperative fatigue in esophageal cancer patients.

Variable	B	SE	*β*	*t*	*P*
Age 1					
<60years	-1.437	0.301	-0.070	-2.660	0.008
herapy method2					
Preoperative neoadjuvant + surgery	-0.588	0.25	-0.76	-2.35	0.020

The independent variable was assigned, with age as the reference group of>80 years old and treatment as the reference group of simple surgery. Model: R2 = 0.124, adjusted R2 = 0.63, F = 2.038, p<0.001.

## 5 Discussion

This study employed symptom network analysis to identify fatigue as the core symptom within the symptom network of postoperative esophageal cancer patients, with choking sensation, weight loss, and sadness serving as key bridge symptoms. Multiple regression analysis further indicated that age and treatment modality were significant influencing factors for postoperative fatigue. However, it must be emphasized from the outset that the adjusted R² of the regression model in this study was low (0.063), indicating that age and treatment modality explained only 6.3% of the variance in fatigue severity. This finding itself carries significant implications: it strongly suggests that biopsychosocial factors beyond demographic characteristics and treatment history play a more dominant role in the development and progression of postoperative fatigue. Therefore, the following discussion will explore the mechanistic underpinnings of the network analysis findings based on existing evidence and reflect critically on the low explanatory power of the statistical model.

### 5.1 Fatigue is a core symptom in the symptom network

The study shows that fatigue is the central symptom in the pain-fatigue-related symptom cluster and is the most common symptom among cancer patients at all stages of treatment. The central role of fatigue within the network suggests that it may not merely be one of many symptoms, but rather a key outward manifestation of the core pathophysiological processes driving the evolution of the entire symptom network. The underlying mechanisms are likely multi-faceted. Firstly, esophagectomy, as a major trauma, can trigger a systemic inflammatory response, releasing pro-inflammatory cytokines such as Tumor Necrosis Factor-alpha (TNF-α) and Interleukin-6 (IL-6). These cytokines can cross the blood-brain barrier, act on the central nervous system, and directly lead to ‘sickness behavior’ or pathological fatigue. Furthermore, fatigue may serve as a common biological basis for its widespread impact on other symptoms (such as pain and low mood). Secondly, the substantial energy demands of surgical trauma, tissue repair, and immune responses, coupled with frequent postoperative inadequate intake due to dysphagia, can create an energy metabolic crisis, potentially exacerbating fatigue at the cellular level. Research (22) has found that the higher the level of fatigue in cancer patients, the poorer their quality of life tends to be. Furthermore, studies have shown that the incidence of fatigue in esophageal cancer patients is 60.8% (23).Trudel-Fitzgerald et al (24) conducted an 18-month symptom monitoring study on cancer patients and found that fatigue is a precursor to depression, insomnia, and pain. However, the gold standard for treating cancer-related fatigue has not been established, possibly due to the multifactorial nature of fatigue symptoms and the lack of understanding of its underlying mechanisms (25).Yan et al (26) Studies show that nursing interventions based on self-efficacy theory can effectively relieve patients’ fatigue and other symptoms and improve their nutritional status after esophageal cancer surgery. Medical staff should focus on fatigue as a symptom management, and reduce its transmission in the symptom network through precise intervention. Currently, a variety of treatments are available for cancer-related fatigue, including exercise therapy, psychological support, medication, and holistic therapies (27, 28). Among these interventions, exercise therapy has shown the most significant effects (29). It can alleviate the overall symptom burden in patients, which can improve the prognosis of esophageal cancer patients, reduce postoperative complications and readmission rates, and enhance their quality of life.

### 5.2 Choking sensation, weight loss, and sadness are bridge symptoms in the symptom network

Bridge symptoms are those that connect two or more symptom clusters within the network, and their strength can be assessed (30). The symptom with the highest bridge strength (31) is identified as the bridge symptom. This study found that among esophageal cancer patients, the bridge strength of choking sensation, weight loss, and sadness ranks in the top three post-surgery, indicating they serve as the primary bridge symptoms linking three symptom clusters in the network.

This study demonstrated that choking sensation has the strongest bridging strength, suggesting that it may function as a core bridging symptom with a wide-ranging impact on other symptoms. The occurrence of globus sensation(a feeling of having a lump or foreign body in the throat) may be related to factors such as anastomotic healing, postoperative placement of a gastrostomy tube, and functional reconstruction. Therefore, clinical interventions should also pay close attention to symptoms closely associated with globus sensation. As a critical bridge symptom, choking sensation’s importance extends beyond local anatomical changes (such as nastomotic edema) to encompass its role in triggering psychological fear and behavioral avoidance. This symptom can initiate a vicious cycle where choking sensation leads to eating anxiety, reduced food intake, weight loss/malnutrition, and consequent fatigue and sadness. Thus, via cognitive-behavioral pathways, choking sensation functionally links somatic, nutritional, and emotional symptom domains. Existing research (31, 32) indicates that interventions targeting bridging symptoms are more effective than those aimed at other types of symptoms. By identifying bridging symptoms and developing specific intervention plans and strategies, symptom management can be achieved more efficiently and accurately. Studies show that early postoperative eating can significantly improve symptoms such as dysphagia and choking in esophageal cancer patients (33). Therefore, healthcare providers should closely monitor patients with choking to promptly identify adverse reactions and other complications. Based on the findings of Liu Hanxue et al (34), swallowing function training can be integrated into the pre-rehabilitation program for esophageal cancer patients, extending from preoperative to post-discharge stages. This can enhance postoperative swallowing function and alleviate dysphagia and choking. The intervention should be combined with nutritional support and oral motor training to effectively improve swallowing function. Weight loss acts as a bridge symptom linking the network. It can be caused by multiple factors, including postoperative fasting, the placement of a nasogastric tube, and postoperative trauma. Weight loss is most strongly associated with symptoms such as dry mouth, shortness of breath, and sadness, and it has the greatest impact. According to a study by Yang Huan et al (35), the analysis of prospective cohort data from a nutrition intervention trial indicates that weight loss is linked to an increased risk of death from esophageal cancer. Additionally, another study (36) found that difficulty swallowing can lead to reduced food intake, resulting in weight loss and malnutrition, and increasing the 5-year mortality rate by 40%.Therefore, healthcare providers should assess patients ‘nutritional risks, monitor their weight and metabolism, and adjust their nutritional plans as needed. They should also pay attention to the continued weight loss in patients with anastomotic fistulas or tumor recurrence, and provide health education and psychological support. Sadness acts as a bridge in the network of symptoms, often resulting from postoperative physical damage, various discomforts, and concerns about the disease’s prognosis, leading to feelings of sadness and distress. Research indicates that if patients are unwilling to communicate or share their inner troubles with family members, it can exacerbate their sadness (37).Therefore, medical staff should continue to pay attention to the psychological situation of patients after surgery, know the root cause of their sadness through communication, regularly assess the psychological status of patients at different stages and intervene, cognitive therapy, relaxation training and music therapy can be used to reduce the psychological burden of patients.

### 5.3 Factors affecting postoperative fatigue in esophageal cancer patients, including age and treatment methods

This study found that age and treatment method significantly influence postoperative fatigue in esophageal cancer patients. Younger patients tend to experience less postoperative fatigue, possibly due to their better physiological reserves, recovery capabilities, and tolerance to treatment. Research (38) indicates that aging leads to a decline in physical function, which can hinder postoperative recovery and limit the patient’s ability to understand and manage their condition. Furthermore, research (39) indicates that elderly patients, due to irreversible physiological decline such as muscle atrophy and cachexia, not only face a higher risk of unplanned readmission after surgery but are also a key factor in postoperative fatigue. Healthcare providers should adopt a stratified intervention strategy to address the factors contributing to postoperative fatigue in esophageal cancer patients. For younger patients, their physiological recovery advantages can be fully utilized through preoperative rehabilitation training and early postoperative activity to promote functional recovery. For elderly patients, close monitoring of vital signs, prevention of complications, and early rehabilitation training are essential. The postoperative fatigue in esophageal cancer patients who have undergone preoperative neoadjuvant therapy combined with surgery may be due to bone marrow suppression, tissue damage, and oxidative stress caused by the neoadjuvant therapy. These factors, along with inflammation from surgical trauma, can lead to energy metabolism disorders, further contributing to fatigue. Research (40) indicates that combining neoadjuvant therapy with surgery can reduce clinical stage, eliminate local and distant metastases, and improve prognosis compared to conventional surgery. However, due to the combined effects of preoperative neoadjuvant therapy and surgical trauma, patients are more prone to fatigue in the short term after surgery. In addition, anemia may have a potential impact on this symptom; surgical trauma and insufficient postoperative nutrient intake can contribute to anemia, which in turn exacerbates fatigue. Therefore, for patients undergoing neoadjuvant therapy combined with surgery, healthcare providers should closely monitor bone marrow suppression, inflammatory responses, and energy metabolism abnormalities, and enhance collaborative management. A comprehensive assessment of their physical condition is necessary to adjust the treatment plan. Systematic interventions include establishing a fatigue management program that spans the entire process, from preoperative rehabilitation to postoperative remote monitoring, and enhancing the training of healthcare providers to effectively reduce postoperative fatigue.

## 6 Limitations

This study surveyed esophageal cancer patients in only two tertiary hospitals in Sichuan Province, which may limit the representativeness of the sample. Future research should broaden the scope of sampling. Additionally, this study only assessed symptoms at the time of discharge and did not track the trajectory of symptom clusters and core symptoms during the home recovery period after surgery. Future work could employ longitudinal designs to clearly delineate the changes in symptoms and core symptoms among postoperative patients from hospitalization to home recovery, and further explore causal relationships among symptoms within longitudinal networks to enhance the efficiency of symptom management, thereby effectively improving patients’ quality of life. A key limitation of this study lies in the limited explanatory power of the statistical model, which failed to incorporate key variables such as nutritional status, social support, and rehabilitation adherence. This constrains a comprehensive understanding of the factors influencing fatigue. Future research should adopt longitudinal designs, recruit larger samples from multiple centers, and utilize more advanced statistical models (such as structural equation modeling). Integrating biomarkers (such as inflammatory markers) with patient-reported outcomes will be essential to reveal the causal mechanisms underlying the dynamic changes in symptom networks, thereby providing robust evidence for developing efficient and precise symptom cluster management strategies.

## 7 Summary

This study conducted a symptom network analysis in patients with esophageal cancer and identified fatigue as the core symptom, while dysphagia served as a bridge symptom. Age and treatment modality were key factors influencing fatigue. Healthcare providers should focus on both core and bridge symptoms during symptom management and implement targeted interventions to develop more precise and efficient management strategies. However, as this study employed a cross-sectional design, it did not explore the dynamic changes in core symptoms over time. Future research should adopt longitudinal designs to further investigate temporal patterns in symptom evolution. It is recommended that clinical nursing staff prioritize monitoring changes in symptom clusters and core symptoms, and accordingly formulate individualized, precise, and efficient symptom cluster management measures. Targeted interventions based on network characteristics should also be developed. Such efforts will contribute to refining postoperative symptom management in esophageal cancer patients and optimizing clinical decision-making.

## Data Availability

The original contributions presented in the study are included in the article/supplementary material. Further inquiries can be directed to the corresponding author.
